# Differential effect of *Pistacia vera *extracts on experimental atherosclerosis in the rabbit animal model: an experimental study

**DOI:** 10.1186/1476-511X-9-73

**Published:** 2010-07-16

**Authors:** Katerina A Marinou, Katerina Georgopoulou, George Agrogiannis, Theodore Karatzas, Dimitrios Iliopoulos, Apostolos Papalois, Achilles Chatziioannou, Prokopios Magiatis, Maria Halabalaki, Nektaria Tsantila, Leandros A Skaltsounis, Efstratios Patsouris, Ismene A Dontas

**Affiliations:** 1 Laboratory of Experimental Surgery and Surgical Research "Christeas Hall", School of Medicine, University of Athens, Greece; 2 Ministry of Rural Development and Food, Centre of Athens Veterinary Institutes, Agia Paraskevi, Attiki, Greece; 3 Division of Pharmacognosy and Natural Products Chemistry, Department of Pharmacy, University of Athens, Greece; 4 1st Department of Pathology, School of Medicine, University of Athens, Greece; 5 2nd Department of Propedeutic Surgery, Laiko Hospital, School of Medicine, University of Athens, Greece; 6 Experimental - Research Center ELPEN Pharma, Pikermi, Greece; 7 1st Department of Radiology, Aretaieion University Hospital, School of Medicine, University of Athens, Greece; 8 Laboratory of Biochemistry, Department of Chemistry, University of Athens, Greece; 9 Laboratory for Research of the Musculoskeletal System, School of Medicine, University of Athens, Greece

## Abstract

**Background:**

Lipid-enriched diets and oxidative stress are risk factors for the development of atherosclerosis. The effects of the methanolic (ME) and cyclohexane (CHE) extracts of the *Pistacia vera *nut, often included in the Mediterranean diet, were studied in the rabbit model of atherosclerosis.

**Methods and results:**

Twenty-four New Zealand White rabbits received atherogenic diet (Control Group), supplemented with ME (Group ME) or CHE (Group CHE) for 3 months. Previously, a GC-MS and a UHPLC LC-DAD-ESI(-)-HRMS/MS method were developed to investigate the extracts' chemical profiles. Blood samples at baseline and monthly determined lipid profile, lipid peroxidation and liver function. The aorta, myocardium and liver were examined histologically at 3 months.

Groups ME and CHE had significantly higher HDL- and non-significantly lower LDL-cholesterol median % changes from baseline than the Control Group. Triacylglycerol was significantly higher in Group CHE vs. Control. MDA values were significantly lower in Group ME vs. Control and CHE. ALT and AST were significantly higher in Group CHE vs. Control. γ-GT was lower in Group ME vs. Control. Aortic intimal thickness was significantly less in Groups ME and CHE vs. Control; Group ME atherosclerotic lesions were significantly less extensive vs. Groups Control and CHE. Only Group CHE had significant liver fatty infiltration.

**Conclusions:**

During short-term administration concomitantly with atherogenic diet, both *P. vera *extracts were beneficial on HDL-, LDL-cholesterol and aortic intimal thickness. The ME additionally presented an antioxidant effect and significant decrease of aortic surface lesions. These results indicate that *P. vera *dietary inclusion, in particular its ME, is potentially beneficial in atherosclerosis management.

## Background

Cardiovascular disease is the leading cause of mortality in the industrialized part of the world with atherosclerosis being its primary manifestation [1]. The development and progression of atherosclerotic lesions has been studied extensively. Many clinical studies have been performed regarding dietary protocols, such as the Seven Countries study on Mediterranean diet which includes olive oil [2]. Other clinical studies have involved pharmaceutical [3] and surgical interventions alone [4], or in combination [5]. Scientific evidence for the pathogenesis and therapy of atherosclerosis during the last century has derived to a large extent from research protocols on animal models.

The rabbit, one of the most important models for the study of atherosclerosis, rapidly responds to the inducement of atherosclerotic lesions through a high cholesterol diet [6]. Several dietary studies have used this model, including the administration of olive or fish oil and different seed extracts or oils, derived from sunflower seeds, peanuts, flaxseeds or hazelnuts [7-10]. An edible nut, consumed either alone or as an ingredient in traditional recipes, is the pistachio nut from the tree *Pistacia vera*, which belongs to the *Anacardiaceae *family and is distributed in central and southeastern Greece, as well as other Mediterranean and Middle East countries. To our knowledge, only *in vitro *studies are published regarding its bioactive effect on the development of atherosclerosis [11]. We, therefore, sought to investigate the effect of *P. vera *cyclohexane (CHE) and methanolic extract (ME) administration on the rabbit experimental model of atherosclerosis on serum biochemistry, as well as aortic, heart and liver lesions.

## Results

In Table 1 the descriptive statistics of the measured parameters of the 3 Groups are shown. Table 2 shows median % changes from baseline of body weight, lipid profile and antioxidant status biochemical parameters and the overall statistical significances. Table 3 shows median % changes from baseline of liver enzymes activity and the overall statistical significances. Table 4 shows morphometric analysis on rabbit aortas.

**Table 1:** Descriptive statistics: mean values ± standard deviation of body weight and biochemical parameters

	Baseline	1^st ^month	2^nd ^month	3^rd ^month
**Group Control**				

**WEIGHT (Kg)**	3.17 ± 0.21	3.46 ± 0.18	3.54 ± 0.27	3.49 ± 0.19
**TC (mg/dL)**	67.88 ± 5.66	649.88 ± 44.4	987.13 ± 95.00	1347.00 ± 149.33
**HDL-C (mg/dL)**	24.62 ± 2.38	62.37 ± 17.20	59.875 ± 20.95	46.37 ± 4.20
**LDL-C (mg/dL)**	23.72 ± 4.82	555.7 ± 46.54	854.62 ± 83.41	1146.60 ± 159.25
**TAG (mg/dL)**	97.63 ± 12.93	159 ± 24.96	363.13 ± 26.28	770.13 ± 68.48
**MDA (nmol/L)**	1.14 ± 0.14	1.74 ± 0.19	2.22 ± 0.16	3.47 ± 0.47
**ALT (IU/L)**	7.75 ± 2.12	15.37 ± 3.46	24.38 ± 3.30	37.25 ± 5.42
**AST (IU/L)**	11.75 ± 2.87	33.38 ± 8.30	42.00 ± 6.05	47.75 ± 6.69
**γGT (IU/L)**	14.25 ± 2.71	47.13 ± 13.42	77.00 ± 15.23	215.13 ± 67.34

**Group ME**				

**WEIGHT (Kg)**	3.09 ± 0.19	3.24 ± 0.26	3.37 ± 0.15	3.40 ± 0.14
**TC (mg/dL)**	55.75 ± 11.67	697.63 ± 75.20	881.25 ± 66.00	1130.5 ± 146.07
**HDL-C (mg/dL)**	19.75 ± 4.65	57.125 ± 23.27	76.875 ± 15.62	57.62 ± 15.26
**LDL-C (mg/dL)**	23.15 ± 9.16	602.82 ± 84.69	740.52 ± 56.36	958.22 ± 146.61
**TAG (mg/dL)**	64.25 ± 9.42	188.38 ± 49.20	319.25 ± 38.53	573.25 ± 63.15
**MDA (nmol/L)**	1.12 ± 0.09	1.99 ± 0.27	2.73 ± 0.33	3.50 ± 0.71
**ALT (IU/L)**	8.25 ± 2.25	20.88 ± 5.05	33.88 ± 4.97	49.88 ± 4.26
**AST (IU/L)**	16 ± 13.53	39.38 ± 15.36	49.63 ± 16.63	59.63 ± 13.8
**γGT (IU/L)**	16.13 ± 3.78	42.5 ± 10.90	127.13 ± 16.37	161.88 ± 66.13

**Group CHE**				

**WEIGHT (Kg)**	3.17 ± 0.22	3.50 ± 0.27	3.50 ± 0.28	3.52 ± 0.37
**TC (mg/dL)**	65.87 ± 11.59	670.13 ± 52.56	979.50 ± 173.68	1477.13 ± 190.83
**HDL-C (mg/dL)**	16.62 ± 4.00	78.75 ± 7.89	87.75 ± 6.60	68.75 ± 17.88
**LDL-C (mg/dL)**	33.22 ± 11.43	543.62 ± 44.61	807.82 ± 170.22	1234.62 ± 181.51
**TAG (mg/dL)**	80.13 ± 13.55	238.75 ± 46.40	419.63 ± 46.55	868.75 ± 224.28
**MDA (nmol/L)**	1.13 ± 0.10	1.97 ± 0.33	2.54 ± 0.41	4.19 ± 1.02
**ALT (IU/L)**	8.25 ± 4.10	23.00 ± 5.55	36.25 ± 6.54	60.87 ± 17.45
**AST (IU/L)**	14.00 ± 4.00	46.12 ± 11.09	57.37 ± 17.68	86.00 ± 8.83
**γGT (IU/L)**	18.50 ± 3.02	43.87 ± 13.44	124.87 ± 12.50	217.37 ± 25.12

Mean values regarding body weight, lipid profile and oxidative stress during the observation period in the three Groups: Control (atherogenic diet), ME (atherogenic diet plus ME), CHE (atherogenic diet plus CHE).

All variables are presented as mean ± SD

**Table 2:** Percent changes of body weight, lipid profile and oxidative stress in rabbit serum

	Group	% change baseline - 1^st ^month	% change baseline - 2^nd ^month	% change baseline - 3^rd ^month
**RABBIT WEIGHT (Kg)**	Control	9.71	12.91	8.99
	ME	5.05	9.72	9.47
	CHE	7.74	11.80	10.76
	Overall Sig	NS	NS	NS

**TC (mg/dL)**	Control	830.55	1351.17	1941.41
	ME	1155.48^a^	1523.27	2018.04
	CHE	865.03^b^	1396.17	2330.76
	Overall Sig	**< 0.0005**	NS	NS

**HDL-C (mg/dL)**	Control	189.74	185.74	81.55
	ME	163.33	262.98^a^	212.42^a^
	CHE	344.74^a b^	350.00^a b^	294.74^a^
	Overall Sig	**< 0.0005**	**< 0.0005**	**< 0.0005**

**LDL-C (mg/dL)**	Control	2124.53	3148.05	4876.60
	ME	2355.51	3239.36	4301.47
	CHE	1524.47^a b^	2403.38^a^	4011.39
	Overall Sig	**< 0.0005**	**< 0.0005**	NS

**TAG (mg/dL)**	Control	56.43	275.45	708.35
	ME	200.00^a^	388.28^a^	810.75
	CHE	188.18^a^	356.43^a^	927.84^a^
	Overall Sig	**< 0.0005**	**< 0.0005**	**< 0.0005**

**MDA (nmol/L)**	Control	50.19	91.93	196.37
	ME	64.71^a^	70.26	160. 51^a^
	CHE	73.71	146.43^b^	297.85^b^
	Overall Sig	**< 0.0005**	**0.032**	**0.012**

Median % change from baseline in body weight, lipid profile and oxidative stress values during the observation period in the three Groups: Control (atherogenic diet), ME (atherogenic diet plus ME), CHE (atherogenic diet plus CHE).

^a ^Significant difference for p < 0.05, compared to Control Group

^b ^Significant difference for p < 0.05, compared to Group ME

Overall Sig: Overall significance, NS: Non-significant

**Table 3:** Percent changes of liver enzyme activities in rabbit serum

	Group	% change baseline-1^st ^month	% change baseline-2^nd ^month	% change baseline-3^rd ^month
**ALT (IU/L)**	Control	105.00	202.14	350.71
	ME	163.89	327.22^a^	448.89
	CHE	198.57^a^	429.76^a^	648.81^a^
	Overall Sig	**0.002**	**0.005**	**0.001**

**AST (IU/L)**	Control	167.53	247.08	300.32
	ME	268.18	415.15	472.73
	CHE	250.77	382.05	569.23^a^
	Overall Sig	NS	NS	**0.018**

**γGT (IU/L)**	Control	231.94	436.90	1345.75
	ME	183.59	650.38^a^	855.06^a^
	CHE	123.68^a^	561.40^a^	1227.86^b^
	Overall Sig	**< 0.0005**	**< 0.0005**	**< 0.0005**

Median liver enzyme activity % change from baseline during the observation period in the three Groups. Control Group: atherogenic diet, Group ME: atherogenic diet plus ME, Group CHE: atherogenic diet plus CHE

^a ^Significant difference for p < 0.05, compared to Control Group

^b ^Significant difference for p < 0.05, compared to Group ME

Overall Sig: Overall significance, NS: Non-significant

**Table 4:** Morphometric analysis on rabbit aortas

Group	thickness (mm)	surface area (mm^2^/mm)
Control (n = 8)	0.883 ± 0.199	0.932 ± 0.161
ME (n = 8)	0.152 ± 0.128^a^	0.256 ± 0.159^a^
CHE (n = 8)	0.468 ± 0.222^a b^	0.864 ± 0.205^b^

Results are expressed as median ± SD.

Control Group: atherogenic diet, Group ME: atherogenic diet plus ME, Group CHE: atherogenic diet plus CHE

^a ^Significant difference for p < 0.05, compared to Control Group

^b ^Significant difference for p < 0.05, compared to Group ME

### Body weight

The final median % change from baseline in Groups Control, ME and CHE was not statistically significant (Table 2), although a mild increase in absolute mean values was observed (Table 1).

### Biochemical values

#### Plasma lipid levels

Plasma lipid levels of all three Groups were similar at baseline. The median % changes from baseline during the observation period described below are shown in Table 2.

##### TC

The median % change from baseline to 1^st ^month for Groups ME and CHE was statistically significantly higher compared to the Control Group (p = 0.01 and 0.05 respectively).

##### HDL-C

The median % change of Group CHE was statistically significantly higher compared to Groups Control and ME at the 1^st ^and 2^nd ^months, while Groups ME and CHE had statistically significant higher values than the Control Group in the 2^nd ^(p = 0.001 and p < 0.005 respectively) and 3^rd ^month (p = 0.01, p < 0.005 respectively).

##### LDL-C

The median % change of Group CHE was statistically significantly lower compared to the Control Group, in the 1^st ^and 2^nd ^month (p < 0.005), while the median % change of Group CHE was statistically significantly lower compared to Group ME only in the 1^st ^month (p = 0.038).

##### TAG

The median % change of Group ME was statistically significantly higher compared to the Control Group in the 1^st ^and 2^nd ^month (p < 0.005 and p = 0.001 respectively). The % change of Group CHE values was significantly higher than those of the Control Group throughout the study (p < 0.005, p = 0.003 and p = 0.05 respectively).

#### Antioxidant evaluation

##### MDA

The median % change of Group CHE was statistically significantly higher compared to Group ME in the 2^nd ^and 3^rd ^month (p = 0.032 and 0.012 respectively). Moreover the median % change of Group ME was statistically significantly different compared to the Control Group in the 1^st ^and 3^rd ^month (p < 0.0005 and p = 0.032 respectively).

#### Liver function

##### ALT

The median % change of Group ME was statistically significantly higher compared to the Control Group in the 2^nd ^month (p = 0.05), while the median % change of Group CHE compared to the Control Group was significantly higher throughout the study (p < 0.05).

##### AST

The median % change of plasma values of all Groups did not show a statistically significant increase during the whole experimental period with Group CHE having higher values only in the 3rd month (p = 0.007).

##### γGT

The median % change of Group ME values was statistically significant lower compared to Groups Control and CHE in the 3^rd ^month of the experiment (p < 0.005).

### Tissue pathology

#### Aorta

**Figure 1:**
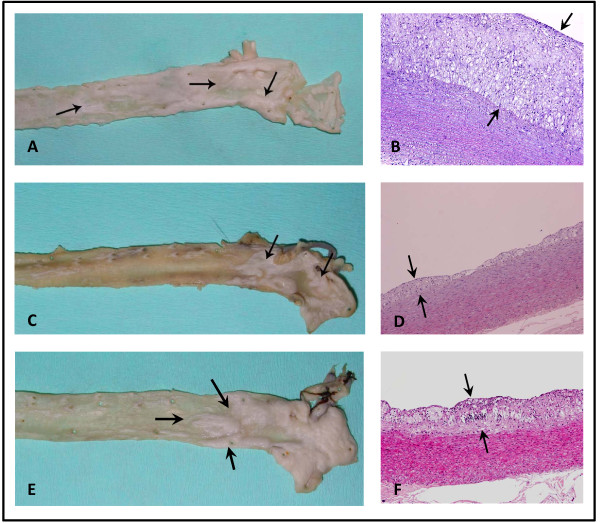
**Macro- and microscopic images of representative aortas from rabbits of the three Groups**. 1A, 1C, 1E are representative images from the gross aortic specimens belonging to Groups Control, ME and CHE, respectively. Arrows indicate atherosclerotic plaque formations. As depicted, the Control Group exhibited extensive atherosclerotic plaques covering almost the whole upper part of the excised aorta. Groups ME and CHE exhibited less extensive lesions compared to Control Group, and between these two groups, Group ME had the least lesion formation. 1B, 1D, 1F are microphotographs from 1A, 1C, 1E respectively (eosin - hematoxylin, original magnification ×100). Figure 1B (of Control Group) shows a vulnerable plaque with many foam cells, inflammation and remarkable intimal thickening. Figure 1D (Group ME) shows the above described lesion, but less extensive (intima/media ratio thickness is less than in 1B. Figure 1F (Group CHE) demonstrates aortic endothelium with evidence of thickening and many foam cells, however the intima/media ratio is less than in 1B. Arrows indicate the width of the endothelium.

Aortic macroscopic specimens from Groups Control and CHE showed extensive atherosclerotic plaques covering almost the whole upper part of the excised aorta (Table 4 and Fig. 1A and 1E). Group ME specimens showed less extensive lesions compared to Group A, as well as compared to Group CHE (Table 4 and Fig. 1C).

Histopathological findings from Group Control and CHE showed vulnerable plaques with many foam cells, inflammation and remarkable intimal thickening (Fig. 1B and 1F). In Group ME the above described lesions, but less extensive, were detected (Fig. 1D).

#### Heart and liver

Histological examination of the heart did not reveal significant changes between Groups.

Histological examination of the liver did not reveal significant changes between Groups Control and ME. Group CHE presented statistically significant fatty infiltration compared to the Control Group.

## Discussion

The non-genetic hyperlipidemic rabbit is an animal model widely used in atherosclerosis studies. Various levels of dietary cholesterol (0.2-1.3%) have been used to create the disease experimentally [12,13] and concomitant administration of other potentially beneficial substances has been studied for periods varying from 8 [12] up to 14 weeks [10,12].

The *Pistacia *plants are trees or bushes indigenous to Greece and other Mediterranean and Middle East countries. Among their 11 species, *P. lentiscus *has been shown to have antioxidant [14], antibacterial [15,16], liver-protective [17] and cytostatic [14] effects and to prevent human LDL-cholesterol from oxidation *in vitro *[18]. *P. terebinthus *has been demonstrated to have a beneficial action on uterine condylomata and skin melanomas [14]. Few studies have been carried out on *P. vera*. It has been reported to have plant antifungal effects [19]. *P. vera *ME contains small quantities of catechin, a polyphenolic flavonoid that shows potentially higher antioxidant activity than ascorbic acid and α- tocopherol in *in vitro *studies [20]. Catechin and its products have also been indicated as factors of reducing cardiovascular risk by lowering serum cholesterol levels, diminishing platelet aggregation and reducing blood pressure [21].

In the present study we investigated the effect of the co-administration of *P. vera*, on the progress of cholesterol-induced atherosclerosis of the rabbit animal model for 12 weeks. We chose to administer the ME or CHE of *P. vera *separately in different animal groups, in order to be able to identify which components would be responsible for the potentially different results. GC-MS analysis of the CHE showed that its major compounds are b-sitosterol, squalene, stigmasterol, oleic and palmitic acid. Clinical trials have already shown the protective effects of such plant sterols in the development of coronary heart disease [22]. Furthermore, analysis of the ME using LC-HRMS/MS techniques revealed the presence of gallic methyl ester, protocatechuic acid and gallic acid. Gallic acid has been demonstrated to have antiatherogenic activity [23].

The results of the present study show a beneficial effect of the co-administration of the *P. vera *ME and CHE in cholesterol enriched diet on HDL-C changes. ME also demonstrated less MDA change from baseline, revealing thus a mild antioxidant effect. The LDL-C beneficial effect of ME was not statistically significant. The other biochemical parameters examined (TC, TAG and liver enzymes), did not demonstrate a beneficial effect of the ME with the analysis of data using % change median values. The analysis of our data using mean absolute values showed a statistically significant protective effect of the ME on the above parameters (Table 1). However, because of the lack of normal distribution of the values, use of the non-parametric analysis (Kruskal-Wallis and Mann-Whitney tests) was deemed necessary.

Several plant substances have a beneficial effect on blood biochemistry of cholesterol-fed rabbits. The olive oil polyphenol known as hydroxytyrosol increased the HDL-C levels of atherogenic diet-fed rabbits [13]. Sesame flour had a beneficial effect on the total cholesterol and triglyceride levels of cholesterol-fed rabbits [24]. Also, secoisolariciresinol diglucoside (SDG), a plant lignan isolated from flaxseed when administered to rabbits for 8 weeks resulted in a decrease of LDL-C and an increase in HDL-C and antioxidant activity [9], similarly to our study. Furthermore, another study in rabbits using ethanol extract of *Hypericum lysimachioides *Boiss var *lysimachioides *(Guttifera), showed a statistically significant decrease of MDA levels, as well as an increase of HDL-C levels, in agreement with our study [25].

Our results showed that treatment with both extracts of *P. vera *induces a mild increase in TC values, although this is not significantly different from the Control Group in the second and third month of administration.

HDL-C median values in the Control Group were lower at the end of the experiment, whereas the co-administration of *P. vera *extracts (Groups ME and CHE) significantly inhibited this decrease. Considering that low HDL-C is associated with a higher risk of cardiovascular disease, it is supported that increased HDL-C will result in a protective effect against atherosclerosis, after prolonged administration of the *P. vera *extracts.

Our results showed significant differences in plasma MDA levels between the ME and the Control Groups. This suggests that the *P. vera *ME's beneficial effect on some lipids and the aorta may be due to a mild anti-oxidant effect.

In our study, liver enzyme levels increased reaching maximum levels the third month in all groups. Specifically, ALT and AST changes were increased in Groups ME and CHE compared to Control. Both clinical and experimental studies have demonstrated that elevated ALT and AST can be predictive for the development of atherosclerosis [26,25]. Furthermore, γGT changes of Group ME were statistically significantly lower in the third month compared to the Control Group. This is indicative of a beneficial effect of the *P. vera *ME and CHE in the function of the biliary system of atherosclerotic animals. In the study by Hakimoglu et al. [25] liver hydropic and lipid degeneration was decreased in ethanol extract of *Hypericum lysimachoides *compared to that of the rabbits fed only with cholesterol enriched diet. This finding is similar to ours, where the liver histology of ME fed rabbits was less affected than the cholesterol group (Control), although there was no statistically significant difference. Specifically, the Control Group rabbits suffered from hepatic damage characterised by steatosis and fibrosis, which was even more evident in Group CHE.

Macroscopically plaque formation in Group ME was less extensive compared to Groups Control and CHE. Similarly to our study, other researchers have observed that despite increase of serum cholesterol after the administration of an extract, the pathology results of the aortic specimens may show an amelioration of the progress of atherosclerosis [27]. Aguilera et al. [8] found that virgin olive oil and fish oils reduce the development of atherosclerotic plaques. Hazelnut oil has also been shown to reduce atherosclerotic lesions in the aorta of cholesterol fed rabbits [10]. In addition, the effect of green tea administration containing epicatechin derived compounds in rabbits showed a beneficial effect on the arterial atherosclerotic plaques [28]. This is in agreement with our findings, which indicated a significant beneficial effect of the *P. vera *ME on the development of aortic atherosclerosis. Specifically, Group ME presented significant inhibition of atherosclerotic plaques regarding both their thickness and extent within the aortic lumen. This may be attributed to the major ME components, such as gallic acid and catechin that may be responsible for this inhibition process as already indicated [22,28]. On the other hand, Group CHE exhibited milder atherosclerosis inhibition regarding plaque thickness, which can be attributed to the presence of b-sitosterol, squalene, stigmasterol, oleic and palmitic acid, as similarly shown [29,30].

Group CHE exhibited the lowest antioxidant activity. This effect can possibly be linked to the extensive but not so complicated atherosclerotic lesions observed in Group CHE aortic specimens compared to those of Groups Control and ME. Group ME, however, exhibited a much stronger antioxidant activity. The statistically significant observed antioxidant effect of the ME, evident by the MDA analysis, concomitantly decreased aortic plaque deposition significantly. On the other hand, the CHE did not affect the extent of atherosclerotic lesions. These findings may be attributed to *P*. vera ME constituents exerting antioxidant activity [11].

## Conclusions

In conclusion, during short-term administration concomitantly with atherogenic diet, both *P. vera *extracts were beneficial on HDL-, LDL-cholesterol and aortic intimal thickness. The ME additionally presented an antioxidant effect and significant decrease of aortic surface lesions. These results indicate that *P. vera *dietary inclusion, in particular its ME, may potentially be beneficial in atherosclerosis management. *P. vera *ME and CHE are being used *in vivo *for the first time to our knowledge in the study of atherosclerosis and show a promising effect regarding its inhibition process. More research is required before *P. vera *may be clinically recommended for dietary inclusion for atherosclerosis management.

## Methods

### Animals

Twenty-four conventional male New Zealand White rabbits (2.7 ± 0.2 Kg), purchased from a Greek approved commercial breeder, were randomly divided into three equal groups (Control, ME and CHE) and kept singly in stainless steel cages with free access to food and tap water. The animal house conditions consisted of 20 ± 2°C, 60 ± 5% relative humidity, under a 12:12 h light: dark cycle. The animals were handled according to standards imposed by the European Directive 86/609/EEC. The local veterinary authorities and animal ethics committee approved (License No. K/950) the study. The Control Group received standard rabbit balanced diet (chemical composition: total fatty acids 2.5%, cellulose 18.5%, total protein 16.5%, water 13%, ash 11%, calcium 1.4%, lysine 0.6%, methionine-cystine 0.55%, phosphorus 0.55%, sodium 0.25%) enriched with 1% cholesterol (Dolder, Switzerland) (atherogenic diet), Group ME received atherogenic diet plus ME (1% v/w) and Group CHE atherogenic diet plus CHE (5% v/w). Diets were freshly prepared every three days before use.

### Preparation and analysis of the extracts

#### Cyclohexane Extract of *P. vera *(CHE)

A quantity of 15 Kg powdered pistachio nuts collected in the Greek island of Aegina was extracted at room temperature, first to be defatted with cyclohexane, giving 7.5 Kg of a green oil residue after evaporation of the solvent. The CHE was saponified by the usual procedure resulting to esterified fatty acids. The fatty acid methylesters of the oils and the unsaponified residue were analyzed by GC and GC/MS to afford b-sitosterol, squalene, stigmasterol, oleic acid and palmitic acid as major compounds (see Additional file 1) .

#### Methanolic Extract of *P. vera *(ME)

After the extraction of powdered pistachio nuts with cyclohexane, the plant material was further extracted with dichloromethane giving 1.5 Kg of green oily extract and then with methanol to give a residue of 500 g after evaporation of the solvent. A quantity of 400 g of the residue was subjected to XAD-4 resin for sugar removal and a ME enriched in phenolic compounds was obtained. In order to describe the chemical profile of the enriched extract a LC-DAD-ESI(-)-HRMS/MS method was developed. The analysis was performed using a UHPLC apparatus connected to the high resolution hybrid LTQ-Orbitrap Discovery spectrometer. The phenolic compounds, gallic methyl ester (**1**), gallic acid (**2**), protocatechuic acid (**3**) catechin (**4**) and epicatechin (**5**) were used as standards for the analysis and the identification thereof was accomplished by comparing the retention time, UV-Vis spectrum and high accurate mass spectra of the peaks in the sample to those of standard compounds.

In the attached Additional file 1 a detailed analysis of the extracts' components is presented.

### Blood samplings and biochemical values

All animals were fasted 12 hours prior to blood sampling. They were mildly sedated (ketamine hydrochloride 12 mg/kg, xylazine 2.5 mg/kg body weight, im) for the procedure in order to avoid stress impact. Blood samples from the auricular artery of animals were placed into Wassermann tubes containing anticoagulant at 0, 1, 2 and 3 months of the experimental procedure. Plasma was separated by centrifugation at 3500 rpm for 15 min. Plasma total cholesterol (TC), high-density lipoprotein cholesterol (HDL-C), low-density lipoprotein cholesterol (LDL-C), triacylglycerol (TAG) concentrations, serum alanine aminotransferase (ALT), serum aspartate aminotransferase (AST) and gamma glutamyl transferase (γGT) activities were measured by commercial enzymatic test kits according to the manufacturer's instructions (Biomerieux, Lyon, France) using an automatic analyser (Type 7170A, Hitachi, Tokyo, Japan). MDA was calculated by the thiobarbituric acid reactive substances (TBARS) manual method as described by Yagi [31]. At the end of the experimental study and after the last blood sampling under sedation, the rabbits were euthanized with sodium thiopental (30 mg/kg iv).

### Tissue samples

The aorta was removed from the aortic arch to the iliac bifurcation. The tissues adhering to the adventitia were removed and the aorta was cut longitudinally along the mid-ventral wall. The aorta was then fixed flatly in 10% phosphate buffered formalin solution. The luminal surface of each aortic specimen was photographed and the image was stored electronically. Sections from all specimens were obtained from three standard sites (immediately distal to the branch of the left subclavian artery, at the seventh intercostal artery and immediately posterior to the celiac artery). These samples were embedded in paraffin blocks and stained with hematoxylin-eosin. The histopathologic atherosclerotic lesions of the aorta were classified according to the Stary [32] classification, while thickness and surface area of atherosclerotic lesions in the wall of aortae were semiquantified using an automated image analysis system [33]. In brief, parameters evaluated were: intimal thickening, foam cell accumulation, mononuclear infiltrates, lipid core and fibrous cap formation. Digital images were obtained from the slides by a photomicroscope (Nikon Eclipse 80i, Nikon Corp, Tokyo, Japan) equipped with a digital camera (Nikon DS - 2 MW). All the images were transferred to a PC with the appropriate software (Image ProPlus v. 5.1, Media Cybernetics, MD, USA).

The heart and liver were weighed and fixed in 10% phosphate buffered formalin solution. Standard sections were taken, embedded in paraffin blocks for hematoxylin-eosin, and the myocardium specimens were additionally stained with Masson's trichrome stain.

Myocardial lesions were graded from 0 to 3 regarding interstitial edema, fibrosis and foam cell infiltrates. Liver lesions were classified in four grades as previously described [34], regarding alterations of architecture, fatty infiltration and fibrosis.

### Data analyses

Data was expressed as mean values ± standard deviation (SD) as well as median values because of violation of normality. The Kolmogorov-Smirnov test was utilized for normality analysis of the parameters.

To indicate the trend in the first 3 months of treatments the median percentage changes after 1, 2 and 3 months of variables respectively were calculated. Comparison of percentage change from baseline of variables during the observation period and the histopathology variables between the three groups were analyzed using the Kruskal-Wallis test and Mann-Whitney test (pairwise comparisons).

All tests were two-sided, statistical significance was set at p < 0.05. All analyses were carried out using the statistical package SPSS vr 16.00 (Statistical Package for the Social Sciences, SPSS Inc., Chicago, Ill., USA).

## Abbreviations

*P.vera: Pistacia vera; *ME: methanolic extract of *Pistacia vera; *CHE: cyclohexane extract of *Pistacia vera; *GC: Gas Chromatography; MS: Mass Spectrometry; UHPLC: Ultra High Performance Liquid Chromatography; LC: Liquid Chromatography; DAD: Diode Array Detector; ESI: Electron Spray Ionization; HRMS: High Resolution Mass Spectrometry; TC: total cholesterol; LDL-C: low-density lipoprotein cholesterol; HDL-C: high-density lipoprotein cholesterol; TAG: triacylglycerol: MDA: malondialdehyde; ALT: alanine aminotransferase; AST: aspartate aminotranferase; γ-GT: gamma glutamyl transferase; TBARS: thiobarbituric acid reactive substances; SD: standard deviation; SPSS: Statistical Package for the Social Sciences; SDG: secoisolariciresinol diglucoside.

## Competing interests

The authors declare that they have no competing interests.

## Authors' contributions

KM carried out the experimental study including general overview of the animals, preparation of the diets, blood sampling, euthanasias and drafted the manuscript. KG and MH prepared the cyclohexane and methanolic extract and the relevant diets, as well as performed their analyses. GA and EP performed and coordinated all stages of tissue sample pathology. TK and DI participated in the removal of tissues and preparation of the manuscript. AP and AC contributed to the study design. PM conducted the design for the production of *Pistacia *extracts. NT contributed to the preparation of the diets and manuscript. LAS and ID conceived the study design, coordinated the experiments and prepared the manuscript. All authors read and approved the final manuscript.

## Supplementary Material

Additional file 1 **Preparation methods of the cyclohexane and methanolic extracts**. Details regarding the methodology of preparation of the cyclohexane and methanolic extracts, including 2 figures. Click here for file
