# Supplementary material for: Differential effect of Pistacia vera extracts on experimental atherosclerosis in the rabbit animal model: an experimental study
Source: Lipids Health Dis. 2010 Jul 16;9:73. doi: 10.1186/1476-511X-9-73 (PMC2917426; doi:10.1186/1476-511X-9-73)
Supplement: Additional file 1 — Preparation methods of the cyclohexane and methanolic extracts. Details regarding the methodology of preparation of the cyclohexane and methanolic extracts, including 2 figures. [file 1476-511X-9-73-S1.DOC]

Additional file 1.

The preparation of cyclohexane and methanolic extracts of *Pistacia vera* was performed according to the following procedure:

1. Cyclohexane Extract of *P. vera* (CHE)

A quantity of 15 Kg powdered pistachio nuts was extracted at room temperature, first to be defatted with cyclohexane, giving 7.5 Kg of a green oil residue after evaporation of the solvent. The cyclohexane extract was saponified by the usual procedure resulting to esterified fatty acids. The fatty acid methylesters of the oils and the unsaponified residue were analyzed by GC and GC/MS to afford b-sitosterol, squalene, stigmasterol, oleic acid and palmitic acid as major compounds.

The chemical analysis performed in the CHE showed that it is composed from the following substances:

A)Unsaponified:

B-sitosterol (46.71%), squalene (17.89%), stigmasterol (14.40%), 9,19 cyclolanost-3-ol, 24 methyl (9.2%), 9,19 cyclolanost-24-en-3-ol (6.44%)

B) Fatty acids composition

Oleic acid (65.07 %), Palmitic acid (16.43%), Stearic acid (2.58%), Linoleic acid (1.48 %), Palmitoleic acid (1.16 %).

*2.* Methanolic Extract of *P. vera* (ME)

After the extraction of powdered pistachio nuts with cyclohexane, the plant material was further extracted with dichloromethane giving 1.5 Kg of green oily extract and then was extracted with methanol to give a residue of 500 g after evaporation of the solvent. A quantity of 400 g of the methanolic extract was subjected to XAD-4 resin and eluted with water with a total flow rate of 5 mL/min for sugar removal and a methanol extract enriched in phenolic compounds was obtained. In order to describe the chemical profile of the enriched extract a LC-DAD-ESI(-)-MS/MS method was developed. The analysis was performed using a UPLC apparatus connected to the high resolution hybrid LTQ-Orbitrap Discovery spectrometer. The phenolic compounds, gallic methyl ester (**1**), gallic acid (**2**), protocatechuic acid (**3**) catechin (**4**) and epicatechin (**5**), purchased from Sigma Aldrich Co. (St. Louis, MO) were used as standards for the analysis and the identification thereof was accomplished by comparing the retention time, UV-Vis spectrum and high accurate mass spectra of the peaks in the sample to those of standard compounds.

Three different sample concentrations of the extract were prepared dissolving 2.0, 5.0 and 10.0 mg in 1.0 mL of 50% MeOH (LC-MS/MS analysis). For the isolated metabolites two different sample concentrations of 0.10 and 0.05 mg/mL in 50% MeOH (LC-MS/MS) were prepared. Both samples were filtered through a 0.45 μm nylon filter before injected to the LC-MS system. LC-DAD-ESI(-)-MS/MS analysis of the enriched extract as well as the analysis of the standards were performed using an Accela LC system (ThermoFinnigan, San Jose, USA) connected to a PDA detector and to a hybrid LTQ-Orbitrap Discovery spectrometer (ThermoFinnigan, San Jose, USA). ESI source was employed for the analysis in negative mode. A Hypersil GOLD column (Thermo Scientific) (50 x 2.1 mm, 1.9 μm) was used. The mobile phase consisted of 0.1 % aqueous formic acid (solvent A) and methanol (solvent B) was used in a linear gradient mode in flow rate of 200 mL/min. The elution conditions used were: initial A-B (95:5); in 8 min A-B (5:95); for 2 min A-B (5:95); back to initial conditions in 0.1 min; and equilibration for 1.9 min. The ESI source was operated at a at sheath gas flow of 30 arb, auxiliary gas flow of 10 arb, ion spray voltage of 3.5 kV and a capillary temperature of 300 ^o^C. For the ms/ms experiments the energy level for CID was kept at 45%. The chromatograms were recorded at 220, 280 and 365 nm by monitoring spectra within a wavelength range of 190-700nm.

Based on the Rt, UV-Vis spectrum (Fig. 2) and the MS/MS spectra (Fig. 3) the phenolics 1-5 were identified in the enriched methanolic extract of *P. vera*. Gallic methyl ester (1) was found to be the major metabolite (A% = 27.5) whereas gallic acid (2) and protocatechuic acid (3) are also present in significant quantities (A% = 4.2 and A% = 5.9, respectively). Catechin (4) and epicatechin (5) were traced in minor quantities (A% = 0.3 and 0.1, respectively).

UV chromatogram. (280 nm)

**1**

**3**

**2**

**4**

**5**

**Fig. S1**

HPLC-DAD profile of the ME *of P. vera* monitored at 280 nm. On the chromatogram are annotated the Rt of the constituents. **1**, gallic methyl ester (Rt = 3.6); **2**, gallic acid (Rt = 1.2); **3**, protocatechuic acid (Rt = 2.4); **4,** catechin (Rt = 3.4) and **5**, epicatechin (Rt = 2.9)

**1**

**2***

**3**

**4 / 5****

**A**


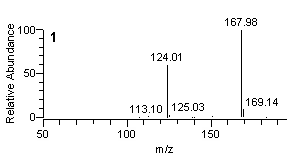


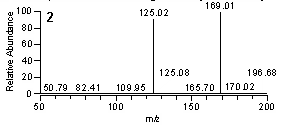


**B**


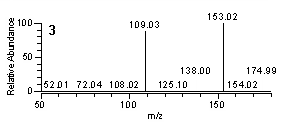

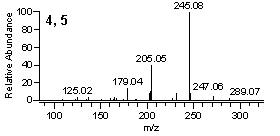


**Fig. S2**

**A** Mass range extracted chromatogram of the ME of *P. vera* for the identified compounds. (**1**) mass extracted chromatogram for gallic methyl ester, (**2**) gallic acid, (**3**) protocatechuic acid, (**4 / 5**) catechin and epicatechin.

**B** MS/MS spectra for compounds (**1**-**5**). CID was kept at 45%.

^*^ peak at Rt = 1.27 corresponds to gallic acid, peak at Rt = 1.97 corresponds to a possible isomer

** catechin (Rt = 3.41) and epicatechin (Rt = 2.96) are epimers giving the same ms/ms spectrum
